# Non-associative versus associative learning by foraging predatory mites

**DOI:** 10.1186/s12898-016-0112-x

**Published:** 2017-01-14

**Authors:** Peter Schausberger, Stefan Peneder

**Affiliations:** 1 Department of Behavioural Biology, University of Vienna, Vienna, Austria; 2 Group of Arthropod Ecology and Behavior, Department of Crop Sciences, University of Natural Resources and Life Sciences, Vienna, Austria

**Keywords:** Associative, Foraging, Learning, Predation, Mites, Non-associative, Thrips

## Abstract

**Background:**

Learning processes can be broadly categorized into associative and non-associative. Associative learning occurs through the pairing of two previously unrelated stimuli, whereas non-associative learning occurs in response to a single stimulus. How these two principal processes compare in the same learning task and how they contribute to the overall behavioural changes brought about by experience is poorly understood. We tackled this issue by scrutinizing associative and non-associative learning of prey, Western flower thrips *Frankliniella occidentalis*, by the predatory mite, *Neoseiulus californicus*. We compared the behaviour of thrips-experienced and -naïve predators, which, early in life, were exposed to either thrips with feeding (associative learning), thrips without feeding (non-associative learning), thrips traces on the surface (non-associative learning), spider mites with feeding (thrips-naïve) or spider mite traces on the surface (thrips-naïve).

**Results:**

Thrips experience in early life, no matter whether associative or not, resulted in higher predation rates on thrips by adult females. In the no-choice experiment, associative thrips experience increased the predation rate on the first day, but shortened the longevity of food-stressed predators, a cost of learning. In the choice experiment, thrips experience, no matter whether associative or not, increased egg production, an adaptive benefit of learning.

**Conclusions:**

Our study shows that both non-associative and associative learning forms operate in foraging predatory mites, *N. californicus*. The non-rewarded thrips prey experience produced a slightly weaker, but less costly, learning effect than the rewarded experience. We argue that in foraging predatory mites non-associative learning is an inevitable component of associative learning, rather than a separate process.

**Electronic supplementary material:**

The online version of this article (doi:10.1186/s12898-016-0112-x) contains supplementary material, which is available to authorized users.

## Background

Learning, changed behaviour following experience, is ubiquitous in animals, from protozoans to primates [1–3]. At large, the huge variety of learning processes can be categorized into non-associative and associative [2, 4]. Associative learning occurs through the association of two previously unrelated stimuli, and includes reinforcement, whereas non-associative learning occurs in response to a single stimulus, without reinforcement. Distinction between these two principal learning categories is not clear-cut and under debate, e.g. [3–6]. Nonetheless, studies rigorously tackling this issue, by, for example, comparing the relative importance of non-associative and associative experiences on the learning success in a given task, such as host or prey recognition, are scarce ([7] for parasitoids). Associative learning involves Pavlovian (classical) and operant (instrumental) conditioning ([8, 9] for honeybees; [10] for cockroaches; [11] for *Drosophila*; [12] for review), while non-associative learning involves sensitization, habituation and imprinting ([1–3] for reviews).

Here, we addressed the behavioural aspects of non-associative vs. associative learning in foraging predatory mites, *Neoseiulus californicus*. *N. californicus* is a plant-inhabiting generalist predator feeding on herbivorous mites such as spider mites and rust and gall mites, small insects such as thrips, and plant-derived substances such as pollen [13–15]. *N. californicus* has a ranked food preference. Among the possible food options, spider mites such as the two-spotted mite *Tetranychus urticae* are the primary prey [13, 16]. Difficult-to-grasp small insects such as larvae of the Western flower thrips *Frankliniella occidentalis* are an alternative, secondary prey, e.g. [17]. *Neoseiulus californicus* has five life stages—egg, larva, protonmyph, deutonymph, adult—and is able to improve its foraging performance by imprinting on a given prey in a sensitive phase early in life, i.e. in the larval and early protonymphal stage [14]. The larvae are, compared to later life stages, little mobile, because of having only six legs, and usually do not feed; the next developmental stage, the protonymph, has eight legs and is the first obligatory feeding stage [18]. The predators are eyeless and use primarily chemo- and mechano-sensory cues to sense their environment, including recognizing suitable prey [19]. For prey, such as thrips, which is difficult to grasp and overwhelm by the fragile small juvenile predators, mere prey contact in early life suffices to establish persistent memory, allowing improving foraging on this prey by the larger adult predators [14]. While food imprinting early in life, a non-associative form of learning [20], produces prey-specific, long-lasting, life stage-crossing effects in foraging *N. californicus* [14], it is unclear how these effects compare to the effects of associative experience made by the predators. Moreover, which prey cues are learned, probably body odours or chemical traces left on the surface, is unknown. These are important issues, from both fundamental and applied perspectives. Studies comparing the operation of different learning processes and their relative contribution to a given learning effect are scarce [7] but inevitable for a thorough mechanistic understanding of learning at the behavioural, perceptual and neuronal levels. Detailed understanding of the learning processes and cues has also relevance to the use of natural enemies, such as *N. californicus*, in biological control, because possibly allowing priming them on a target pest ([21, 22] for parasitoids).

We conducted two experiments, no-choice and choice, to determine which features of the alternative prey Western flower thrips, *F. occidentalis*, are learned by *N. californicus* early in life, and to compare the effects produced by non-associative and associative experience. The prey cues presented to young predatory mites during the learning phase varied in complexity and information content, ranging from (1) prey traces left on the surface, to (2) prey traces left on the surface plus chemical, behavioural and morphological traits on the body of live prey, to (3) prey traces left on the surface plus chemical, behavioural and morphological traits on the body of live prey plus dead prey allowing easy feeding. Treatments (1) and (2) represent non-associative learning paradigms, while treatment (3) represents an associative learning paradigm, because of involving tasting, feeding and/or satiation rewards.

## Results

### No-choice experiment

**Fig. 1:**
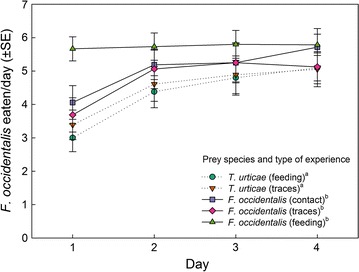
Predation on first larvae of thrips *F. occidentalis* by gravid thrips-experienced and -naïve (spider mite *T. urticae*-experienced) *N. californicus* females over time, in dependence of the predators’ type of experience early in life. Type of experience was either contact with live prey but no feeding (contact), feeding on prey (feeding), or contact with prey traces left on the surface (traces). *Different superscript letters* accompanying prey species and type of experience indicate significant differences (GEE; *P* < 0.05)

**Fig. 2:**
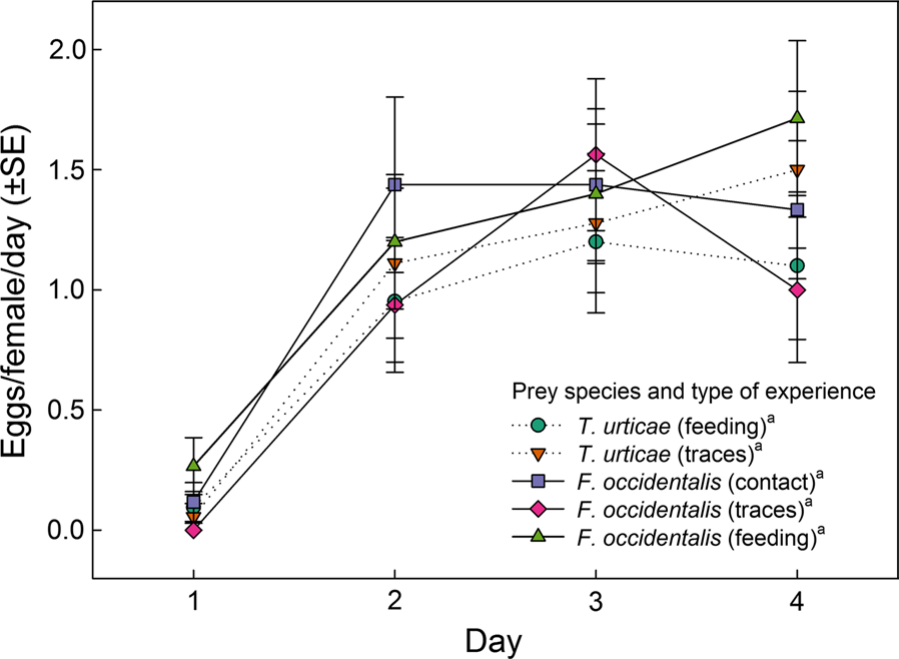
Oviposition by gravid thrips-experienced and -naïve (spider mite *T. urticae*-experienced) *N. californicus* females offered first larvae of thrips *F. occidentalis* as prey over time, in dependence of the predators’ type of experience early in life. Type of experience was either contact with live prey but no feeding (contact), feeding on prey (feeding), or contact with prey traces left on the surface (traces). The *same superscript letter* accompanying prey species and type of experience indicates non-significance (GEE; *P* > 0.05)

**Fig. 3:**
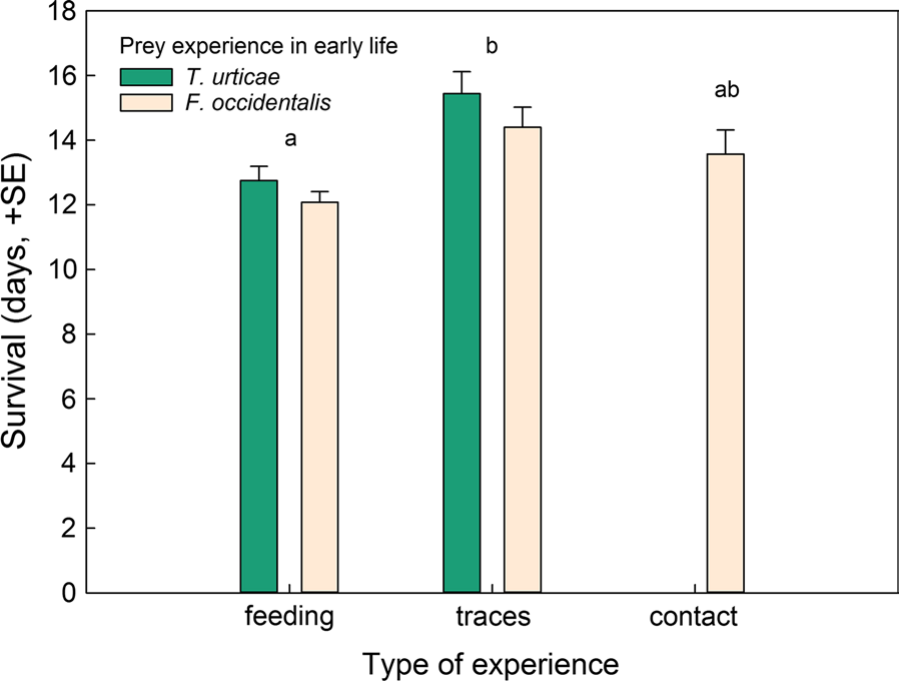
Survival of gravid thrips-experienced and -naïve (spider mite *T. urticae*-experienced) *N. californicus* females offered first larvae of thrips *F. occidentalis* as prey, in dependence of the predators’ type of experience early in life. Type of experience was either contact with live prey but no feeding (contact), feeding on prey (feeding), or contact with prey traces left on the surface (traces). *Different letters* on *top of bars* indicate significant differences among types of experience (Bonferroni following GLM; *P* < 0.05)

Thrips experience (GEE: *Wald ӽ*_*1*_^*2*^ = 5.56, *p* = 0.02) but not type of experience (*Wald ӽ*_*2*_^*2*^ = 1.67, *p* = 0.43) and the interaction between thrips and type of experience (*Wald ӽ*_*1*_^*2*^ = 2.22, *p* = 0.14) affected the number of thrips killed and sucked out over the 4 days experimental period (Fig. 1). Thrips-experienced predators killed more thrips than thrips-naïve predators. Only on the 1st day, associative thrips learners, i.e. those that had experienced thrips by feeding, killed and sucked out more thrips than non-associative thrips learners and thrips-naïve predators (Bonferroni, *p* < 0.05 for each pairwise comparison; Fig. 1). The number of eggs produced did neither vary with thrips experience (GEE: *Wald ӽ*_*1*_^*2*^ = 0.47, *p* = 0.49) nor type of experience (*Wald ӽ*_*2*_^*2*^ = 0.27, *p* = 0.87) nor their interaction (*Wald ӽ*_*1*_^*2*^ = 2.46, *p* = 0.12) over time (Fig. 2). Type of experience affected predator longevity (GLM: *Wald ӽ*_*2*_^*2*^ = 19.61, *p* < 0.001), no matter of thrips experience (*Wald ӽ*_*1*_^*2*^ = 2.29, *p* = 0.13) and the interaction between thrips and type of experience (*Wald ӽ*_*1*_^*2*^ = 0.12, *p* = 0.74) (Fig. 3). Predators that had only experienced prey traces survived longer than predators with feeding experience (Bonferroni: *p* < 0.05); longevity of predators that had contacted prey was intermediate, but not statistically separable (*p* > 0.05 in pairwise comparisons) from longevity of predators experienced with prey traces and those with feeding experience (Fig. 3).

### Choice experiment

**Fig. 4:**
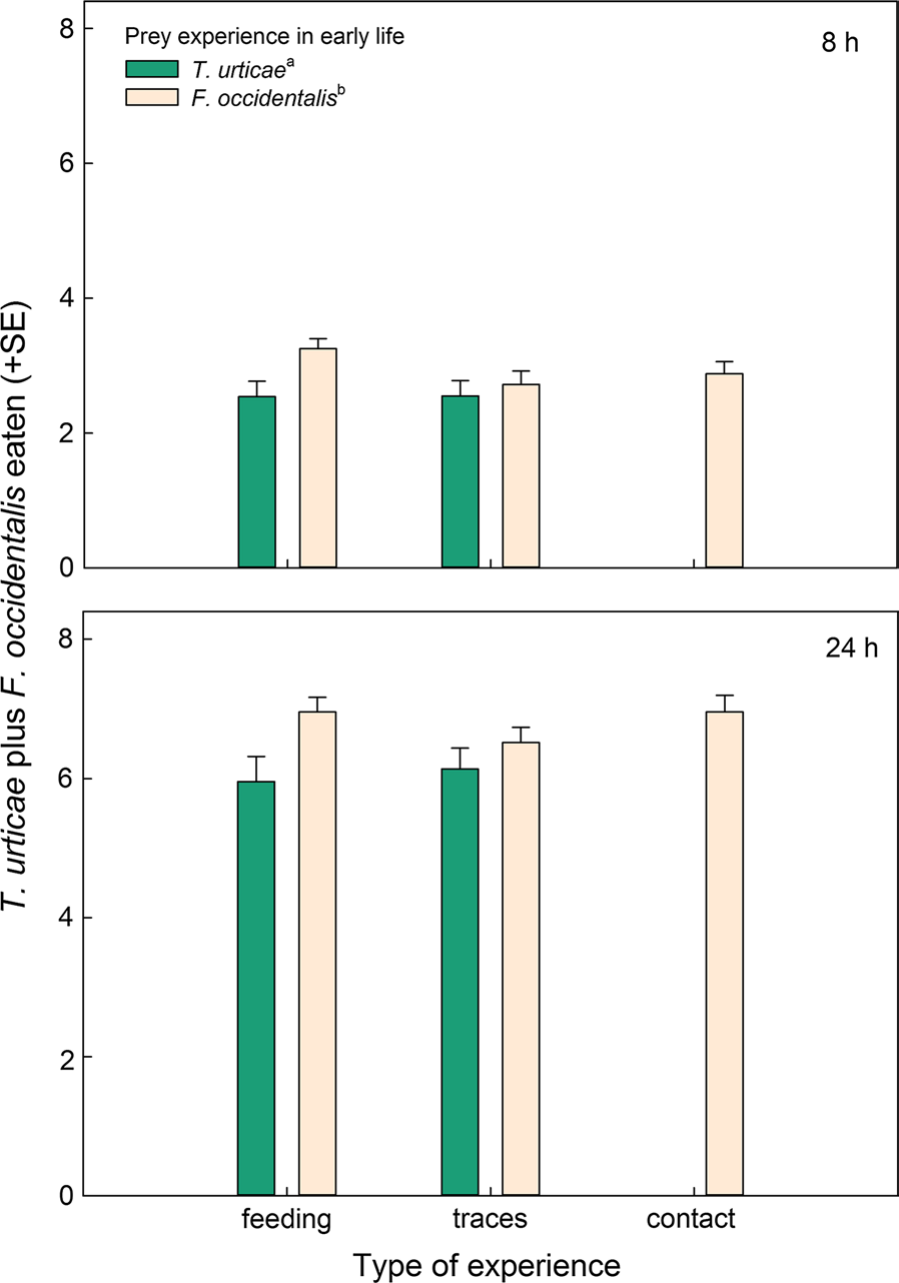
Total number of prey consumed by gravid thrips-experienced and -naïve (spider mite *T. urticae*-experienced) *N. californicus* females simultaneously offered four spider mite nymphs, *T. urticae*, plus four first larvae of thrips, *F. occidentalis*, after 8 and 24 h, in dependence of the predators’ type of experience early in life. Type of experience was either contact with live prey but no feeding (contact), feeding on prey (feeding), or contact with prey traces left on the surface (traces). *Different superscript letters* accompanying prey species experience indicate a significant difference (GEE; *P* < 0.05)

**Fig. 5:**
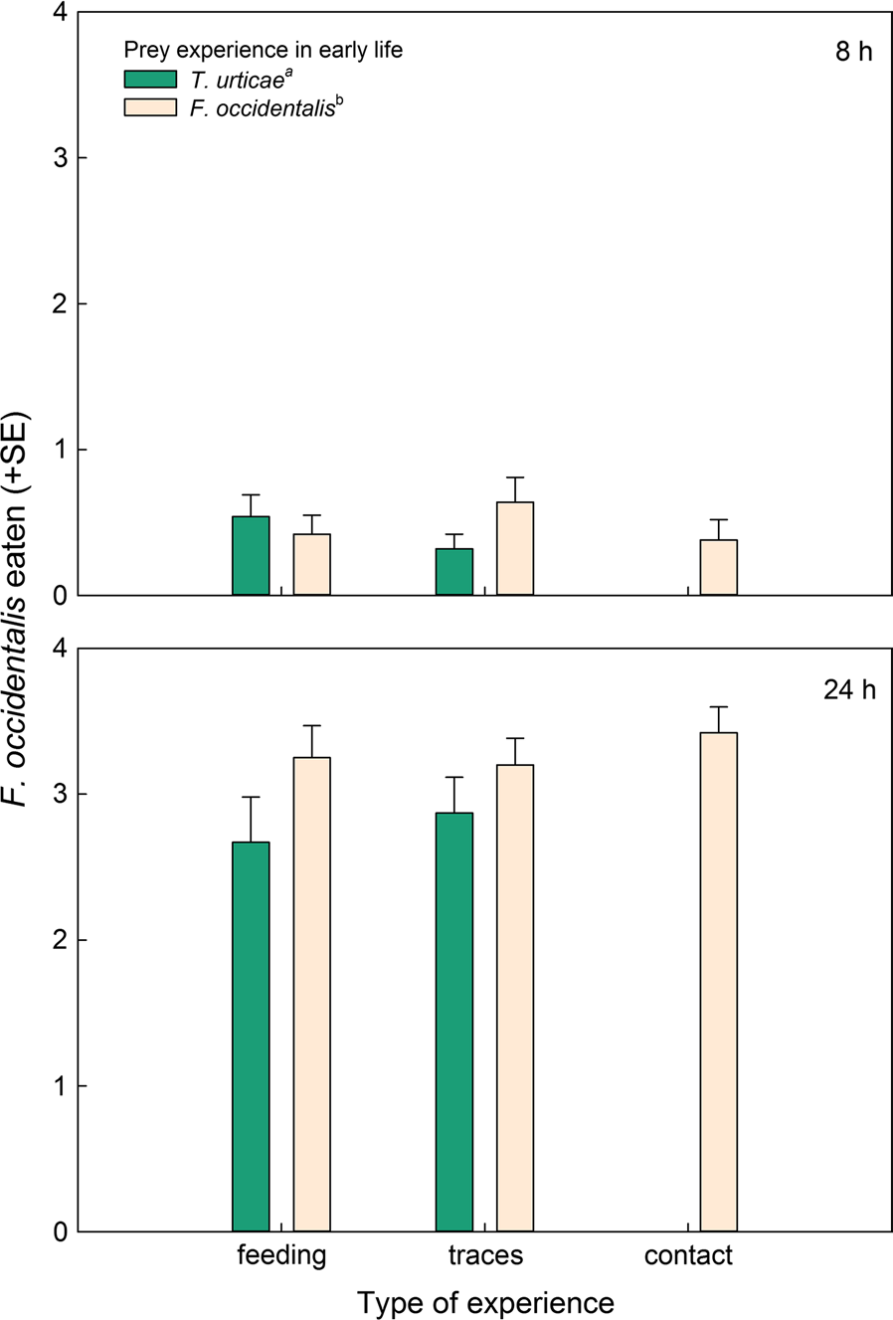
Number of first larvae of thrips, *F. occidentalis*, consumed by gravid thrips-experienced and -naïve (spider mite *T. urticae*-experienced) *N. californicus* females, simultaneously offered four spider mite nymphs, *T. urticae*, plus four thrips larvae, *F. occidentalis*, after 8 and 24 h, in dependence of the predators’ type of experience early in life. Type of experience was either contact with live prey but no feeding (contact), feeding on prey (feeding), or contact with prey traces left on the surface (traces). *Different superscript letters* accompanying prey species experience indicate a significant difference (GEE; *P* < 0.05)

**Fig. 6:**
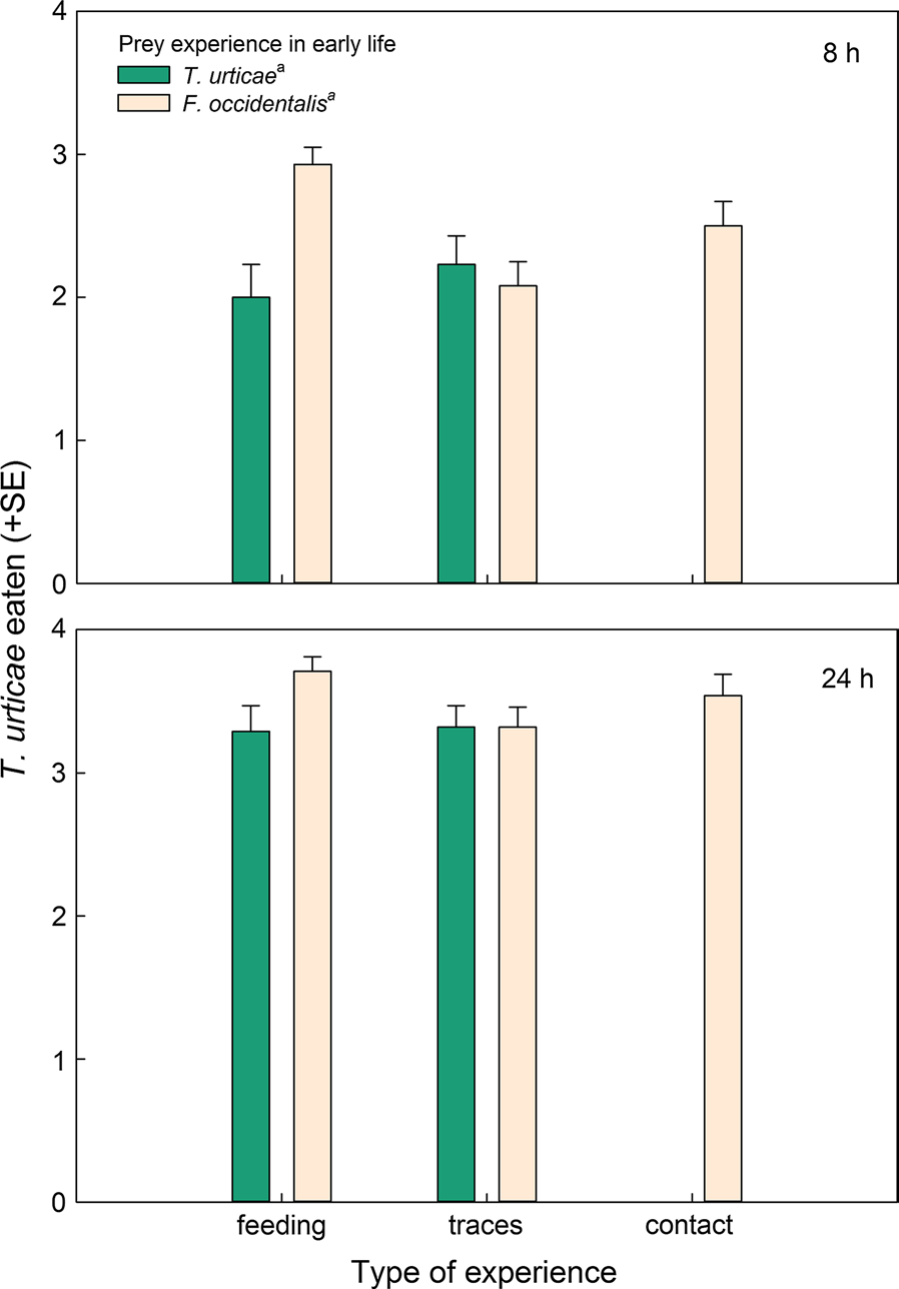
Number of *T. urticae* nymphs consumed by gravid thrips-experienced and -naïve (spider mite *T. urticae*-experienced) *N. californicus* females, simultaneously offered four spider mite nymphs, *T. urticae*, plus four thrips larvae, *F. occidentalis*, after 8 and 24 h, in dependence of the predators’ type of experience early in life. Type of experience was either contact with live prey but no feeding (contact), feeding on prey (feeding), or contact with prey traces left on the surface (traces). The *same superscript letter* accompanying prey species experience indicates non-significance (GEE; *P* > 0.05)

**Fig. 7:**
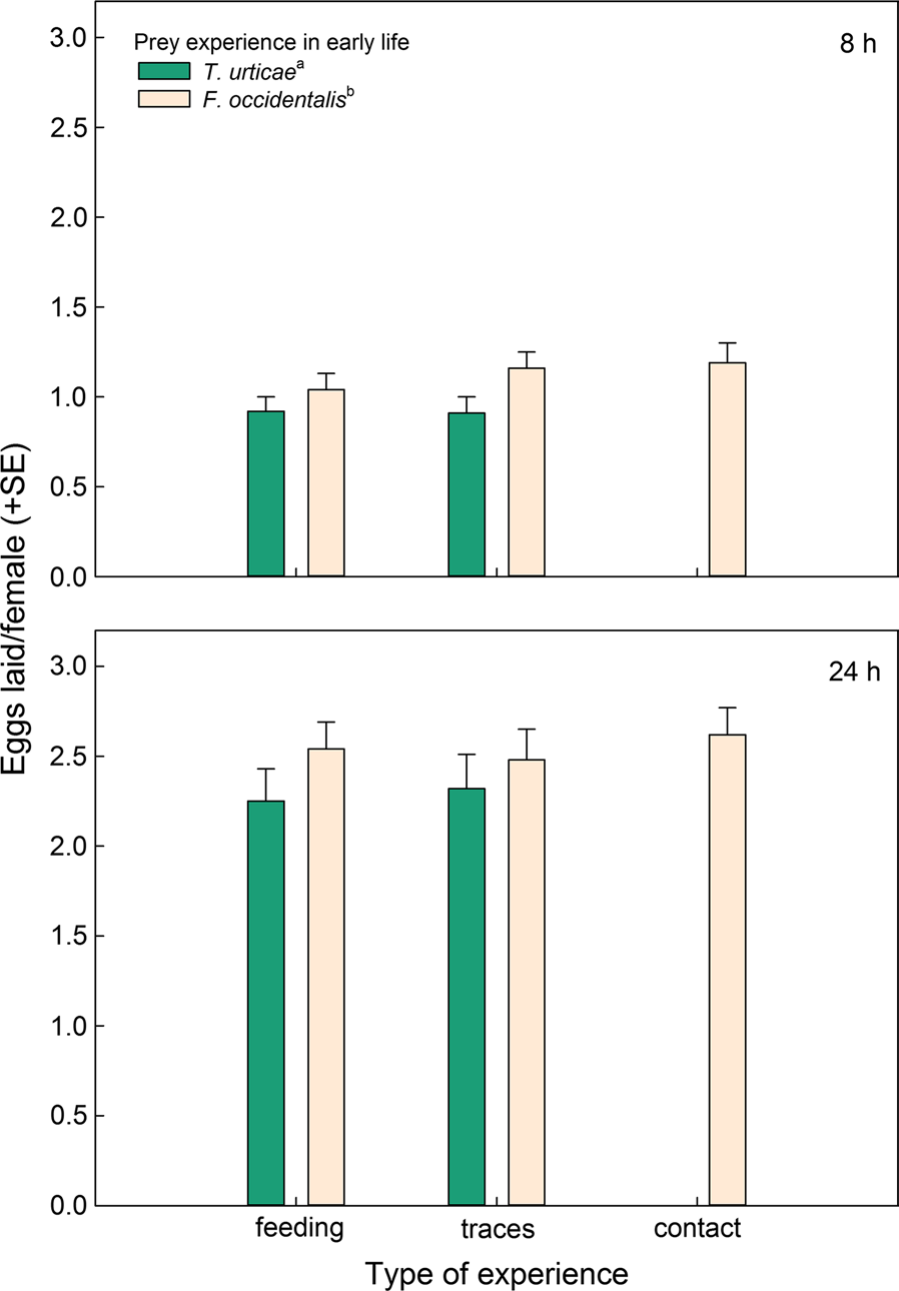
Number of eggs laid by gravid thrips-experienced and -naïve (spider mite *T. urticae*-experienced) *N. californicus* females, simultaneously offered four spider mite nymphs, *T. urticae*, plus four first larvae of thrips, *F. occidentalis*, within 8 and 24 h, in dependence of the predators’ type of experience early in life. Type of experience was either contact with live prey but no feeding (contact), feeding on prey (feeding), or contact with prey traces left on the surface (traces). *Different superscript letters* accompanying prey species experience indicate a marginally significant difference (GEE; *P* = 0.06)

Thrips-experienced predators consumed more prey in total (spider mites plus thrips) than thrips-naïve predators (GEE: *Wald ӽ*_*1*_^*2*^ = 7.69, *p* = 0.006), no matter of the type of experience (*Wald ӽ*_*2*_^*2*^ = 0.86, *p* = 0.65) and the interaction between thrips and type of experience (*Wald ӽ*_*1*_^*2*^ = 1.87, *p* = 0.17) (Fig. 4). This was primarily due to thrips-experienced predators consuming more thrips than thrips-naïve predators (GEE: *Wald ӽ*_*1*_^*2*^ = 5.16, *p* = 0.02), no matter of the type of experience (*Wald ӽ*_*2*_^*2*^ = 0.06, *p* = 0.97) and the interaction between thrips and type of experience (*Wald ӽ*_*1*_^*2*^ = 0.22, *p* = 0.64) (Fig. 5). In contrast, predation on spider mites did neither vary with thrips experience (GEE: *Wald ӽ*_*1*_^*2*^ = 1.14, p = 0.29) nor type of experience (*Wald ӽ*_*2*_^*2*^ = 0.72, p = 0.70) nor their interaction (*Wald ӽ*_*1*_^*2*^ = 1.89, p = 0.17) (Fig. 6). Egg production was marginally significantly higher in thrips-experienced than -naïve predators (GEE: *Wald ӽ*_*1*_^*2*^ = 3.37, *p* = 0.06), no matter of the type of experience (*Wald ӽ*_*2*_^*2*^ = 0.61, *p* = 0.74) and the interaction between thrips and type of experience (*Wald ӽ*_*1*_^*2*^ = 0.00, *p* = 0.99) (Fig. 7).

## Discussion

Our study reveals that both non-associative and associative learning processes operate in foraging predatory mites *Neoseiulus californicus*. Mere contact with the prey *F. occidentalis* or its traces left on the surface was sufficient for learning and establishing long-lasting (considering the predators’ longevity of ~50 days at 25 °C) memory [14]. However, reinforcement of the prey experience made early in life, by pairing external prey cues with a feeding reward (taste and/or satiation), strengthened or intensified the learning effect, as indicated by the initially higher predation rate of predators with thrips feeding experience than those with only thrips contact or traces experience in the no-choice experiment. At the behavioural level, this could mechanistically represent non-associative learning plus the added associative effect, or graded intensities of one and the same learning process (i.e., increasing intensity with increasing cue variety and/or quality), or completely distinct processes. The latter is unlikely because, chronologically, first, orientation on, and recognition of, external prey cues is required, in both non-associative and associative learning, and only then, after recognition and acceptance as suitable prey, in associative learning gustatory cues and satiation come into play, reinforcing learning through the feeding reward. In principle, every associative experience can have, or can build on, components of non-associative learning but this has rarely been assessed ([7] for parasitoids).

At the neuronal level, associative learning may either strengthen or intensify the changes in the same neuronal pathways, as compared to non-associative learning, or establish additional interconnected or separate pathways than non-associative learning. The latter is true for the distinction between short- and long-term memory ([23] for *Drosophila*; [24] for honey bees), which, at the molecular level, trigger differing protein syntheses ([25, 26] for reviews). While in honey bees, non-associative and associative learning could be reflected in different memory retention times [27, 28], this is not applicable to predatory mites, because also non-associative imprinting may produce long-lasting effects [14]. At the genetic level, short- and long-term memory, and possibly non-associative and associative learning, may involve genetically distinct, functionally different components (e.g. [29] for *Drosophila*). At the epigenetic level, short- and long-term memory may be discernible in methylation of learning-related genes (e.g. [30, 31] for honey bees), which may also be true for epigenetic marks produced by non-associative and associative experiences. For predatory mites, any evidence of the involvement of different genes and/or differing epigenetic regulation of non-associative and associative learning remains elusive until identification of learning-associated genes.

Apart from feeding experience increasing the initial predation rate on thrips in the no-choice experiment, we did not observe any differences between the types of experience within thrips-experienced predators. However, prey feeding experience early in life, no matter whether thrips or spider mites, had an effect on survival of the experimental animals, that is, it shortened predator longevity. This might represent an operating cost of associative learning, i.e., a trade-off between learning and life history traits [32, 33]. Energy needed to form new, or strengthen existing, neuronal connections and pathways, was traded off against energy used for basic physiological maintenance and processes, no matter whether the predators then received the prey experienced early in life or a novel prey. Predation rate over time was higher in thrips-experienced than -naïve predators but did not differ among types of thrips-experience (traces vs. contact vs. feeding) in the no choice-experiment. Lacking difference among types of experience within prey species was also evident in the choice experiment. Similar to the no-choice experiment, thrips-experienced predators consumed more thrips and laid more eggs than thrips-naïve predators, no matter of the type of thrips experience (traces vs. contact vs. feeding). Higher egg production of thrips-experienced than thrips-naïve predators points at the adaptive benefits of thrips learning [33]. The choice experiment underlines that the behavioural changes brought about by thrips experience early in life are thrips specific and not the result of unspecific sensitization [14, 33]. If thrips experience in early life would have sensitized the predators, thrips-experienced predators should have fed more on any prey, including spider mites, which was not the case.

At the perceptual level, two or three chemosensory modalities were involved in associative learning, (1) volatile and/or (2) tactile chemoreception and (3) gustation by ingestion, whereas it was just one or two, (1) volatile and/or (2) tactile chemoreception, in non-associative learning. In associative learning, satiation came as an additional internal stimulus into play. Knowledge about the sensory modalities and prey cues playing a role in learning has relevance for the use of *N. californicus,* and other natural enemies, in biological control. Commonly, these predators are mass-reared on other than target prey, possibly compromising their performance against the target pest after release in the crop. Adding chemical extracts or dead corpses from target prey to the mass rearing might enhance the efficacy of the predators, at least in the short term after release. Pertinent proofs of concept are available for parasitoids [21, 22]: parasitoids primed on the target host during rearing performed better against this host in the field than target host-naïve parasitoids.

## Conclusions

Learning processes can be broadly categorized into non-associative and associative but how these two processes compare in the same learning task is poorly understood. We tackled this issue by investigating the effects produced by non-associative and associative learning in plant-inhabiting predatory mites *Neoseiulus californicus* in foraging contexts. Adult predatory mite females memorized, after three molting events, prey, Western flower thrips *Frankliniella occidentalis*, experienced in early life. Associative, rewarded experience produced slightly stronger, but physiologically more costly, learning effects than non-associative experience. Both learning processes resulted in persistent memory. We argue that non-associative learning is an inevitable component of associative learning rather than a completely distinct process.

## Methods

### Predator and prey rearing

*Neoseiulus californicus* used in experiments derived from a laboratory population founded with specimens obtained from Koppert (NL). The predators were reared in piles of detached leaves of common bean, *Phaseolus vulgaris* L., infested by two-spotted spider mites, *Tetranychus urticae* Koch. Detached leaves were piled up on an artificial arena consisting of an acrylic tile (15 × 15 cm) resting on a water-saturated foam cube kept in a plastic box (20 × 20 × 6 cm) half-filled with water. Moist tissue paper was folded over the edges of the tile to prevent the mites from escaping. To obtain predator eggs used for experiments, gravid females were randomly withdrawn from the population, transferred to detached leaf arenas (called oviposition arenas) and provided with mixed *T. urticae* stages. Oviposition arenas consisted of bean leaves placed upside down on water-saturated foam cubes kept in plastic boxes half-filled with water. Moist tissue paper was wrapped around the stem of the leaf to maintain leaf turgidity, and folded over its edges to prevent mite escaping. Eggs laid by the predator females were collected after 24 h for use in experiments. The predator rearing unit, leaf arenas and experimental units were kept in an environmental chamber at 25 ± 1 °C, 60 ± 5% RH and 16:8 h (light:dark) photoperiod.

*Tetranychus urticae* nymphs used as prey in experiments were randomly collected from a population reared on whole bean plants, *P. vulgaris*. Western flower thrips, *Frankliniella occidentalis* (Pergande), was reared on detached bean leaves embedded in 1% water agar (Fluka, Vienna) in closed plastic Petri dishes (14.5 cm diameter). The Petri dish lids were perforated but closed by gauze for ventilation. Nescofilm^®^ was used to tightly connect the lids and the bottom parts to prevent thrips escaping. To obtain first larval stages used as prey in experiments, adult female thrips were transferred to separate detached bean leaves and allowed to lay eggs for 24 h. Every 24 h, the females were transferred to a new leaf. First larvae emerged after 4–6 days and were then used in experiments. Petri dishes were kept in an environmental chamber at 25 ± 1 °C, 60 ± 5% RH and 16:8 h (light:dark) photoperiod.

### Pre-experimental procedures

Predator eggs (<24 h old), giving rise to the experimental animals, were randomly withdrawn from the predator oviposition arenas and placed inside acrylic cages. Each acrylic cage consisted of a circular cavity (∅1.5 cm) laser-cut into an acrylic plate, covered on the bottom side by gauze and on the upper side by a removable microscope slide [34]. The cages were checked daily for hatching larvae, which were then singly transferred to new acrylic cages for the learning phase (dubbed learning cages).

Before placing the predator larvae into the learning cages, the cages were prepared according to one of five treatments, three of which generated thrips-experienced predators and two of which generated thrips-naïve predators. For the three treatments used to generate thrips-experienced predators, each cage received three first larvae of *F. occidentalis* for 24 h. Before adding the predatory mite larva to the cage for the 24 h conditioning phase, for treatment 1 (thrips feeding) one prey larva was left alive and two were killed immediately before, for treatment 2 (thrips contact) all three prey larvae were left alive, and for treatment 3 (thrips traces) all three prey larvae were removed so that only their traces, such as metabolic waste products, remained in the cage. For the two treatments 4 and 5, which were used to generate thrips-naïve predators, each cage received three nymphs of *T. urticae* for 24 h. Before adding the predatory mite larva to the cage, for treatment 4 (spider mite feeding) all three prey nymphs were left alive, and for treatment 5 (spider mite traces) all three prey nymphs were removed immediately before so that only their webbing and traces, such as metabolic waste products, remained in the cage. A treatment “spider mite contact” could not be established because the predators inevitably attack, kill and feed on the spider mites upon encounter. Predators that had possibly fed on thrips in the thrips contact group, determined when a dead thrips was found after 24 h, were discarded. Predators of treatment (1) were considered associative thrips learners, predators of (2) and (3) were considered non-associative thrips learners and those of treatments (4) and (5) were thrips-naïve. After the 24 h learning phase, the predator larvae (or freshly moulted protonymphs) were removed and singly transferred into cages containing mixed spider mite stages as prey (replenished as needed) and left there until reaching adulthood, lasting three to four days. Feeding on thrips by the associative learners in treatment (1) and feeding on spider mites in treatment (4) was verified by the coloured content of the digestive tract of the predators.

### No-choice experiment

Upon reaching adulthood, the predator females were singly transferred to cages that had been previously loaded with seven live first larvae of *F. occidentalis*; a male, randomly withdrawn from the rearing, was added for mating, and the cages checked for the occurrence and number of killed and sucked out thrips larvae after 24 h. After 24, 48 and 72 h the now gravid predator females were singly transferred to new cages, each containing seven thrips larvae, and the number of killed and sucked out thrips larvae and eggs laid by the predators counted, and removed, the next day. Following the fourth thrips counting, i.e., 96 h after starting the experiment, the predators were left in their cages, without replenishing prey, and their survival checked once per day until natural death. Each of the five treatments was replicated 15–21 times.

### Choice experiment

To start the choice experiment, gravid females conditioned and raised according to one of the five treatments described in the pre-experimental procedures, i.e. (1) thrips feeding, (2) thrips contact, (3) thrips traces, (4) spider mite feeding and (5) spider mite traces, were singly placed inside acrylic cages containing four spider mite nymphs plus four first larvae of thrips. The numbers of killed and sucked out spider mites and thrips, and eggs laid by the predators were assessed after 8 and 24 h. Each of the five treatments was replicated 22–26 times.

### Statistical analyses

We used IBM SPSS 23 (IBM Corp., USA) for all statistical analyses. The raw data of both experiments, no-choice and choice, are provided in Additional File 1: Table S1. In the no-choice experiment, we used separate generalized estimating equations (GEE; Poisson distribution, log link) to analyse the influence of thrips experience and type of experience (traces, contact, feeding) on the predation rate on thrips and egg production with thrips prey over the 4 days experimental period (used as auto-correlated inner subject variable). A generalized linear model (GLM; normal distribution, identity link) was used to compare post-experimental survival as affected by thrips and type of experience. In the choice experiment, we used separate generalized estimating equations (GEE) to analyse the influence of thrips experience and type of experience (traces, contact, feeding) on total predation rate (spider mites plus thrips), predation on thrips and predation on spider mites after 8 and 24 h (Poisson distribution, log link; time used as inner subject variable), and eggs laid by the predators within 8 and 24 h (normal distribution, identity link; time used as inner subject variable).

## Additional file

**Additional file 1: Table S1.** Raw data of both experiments, no-choice and choice.
